# Self Organizing Map-Based Classification of Cathepsin k and S Inhibitors with Different Selectivity Profiles Using Different Structural Molecular Fingerprints: Design and Application for Discovery of Novel Hits

**DOI:** 10.3390/molecules21020175

**Published:** 2016-01-30

**Authors:** Saleh K. Ihmaid, Hany E. A. Ahmed, Mohamed F. Zayed, Mohammed M. Abadleh

**Affiliations:** 1 Pharmacognosy and Pharmaceutical Chemistry Department, College of Pharmacy, Taibah University, P. O. Box 30039, Al-Madinah Al-Munawarah 41477, Saudi Arabia; saleh_ihmaid@yahoo.com.au (S.K.I.); mfzayed25@yahoo.com (M.F.Z.); mommetto@hotmail.com (M.M.A.); 2 School of Pharmacy and Applied Science, La Trobe University, P. O. Box 199, Bendigo 3552, Australia; 3 Pharmaceutical Organic Chemistry Department, Faculty of Pharmacy, Al-Azhar University, P. O. Box 11651, Cairo 11884, Egypt; 4 Pharmaceutical Chemistry Department, Faculty of Pharmacy, Al-Azhar University, P. O. Box 11651, Cairo 11884, Egypt

**Keywords:** cathepsin inhibitors, fingerprints, selectivity, self-organizing map (SOM), clustering

## Abstract

The main step in a successful drug discovery pipeline is the identification of small potent compounds that selectively bind to the target of interest with high affinity. However, there is still a shortage of efficient and accurate computational methods with powerful capability to study and hence predict compound selectivity properties. In this work, we propose an affordable machine learning method to perform compound selectivity classification and prediction. For this purpose, we have collected compounds with reported activity and built a selectivity database formed of 153 cathepsin K and S inhibitors that are considered of medicinal interest. This database has three compound sets, two K/S and S/K selective ones and one non-selective KS one. We have subjected this database to the selectivity classification tool ‘Emergent Self-Organizing Maps’ for exploring its capability to differentiate selective cathepsin inhibitors for one target over the other. The method exhibited good clustering performance for selective ligands with high accuracy (up to 100 %). Among the possibilites, BAPs and MACCS molecular structural fingerprints were used for such a classification. The results exhibited the ability of the method for structure-selectivity relationship interpretation and selectivity markers were identified for the design of further novel inhibitors with high activity and target selectivity.

## 1. Introduction

**Figure 1:**
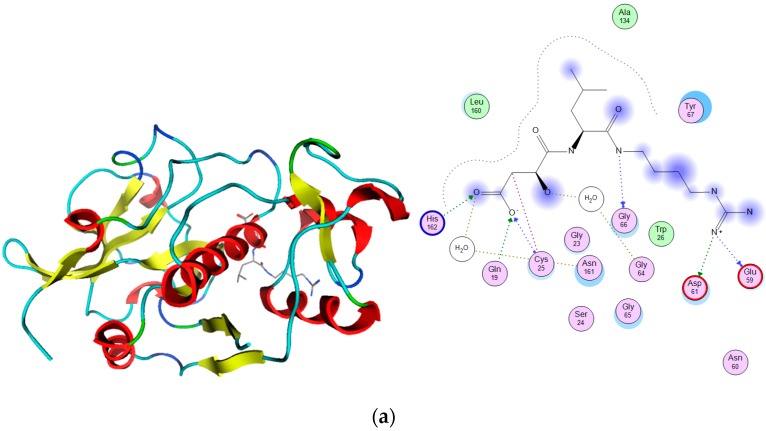

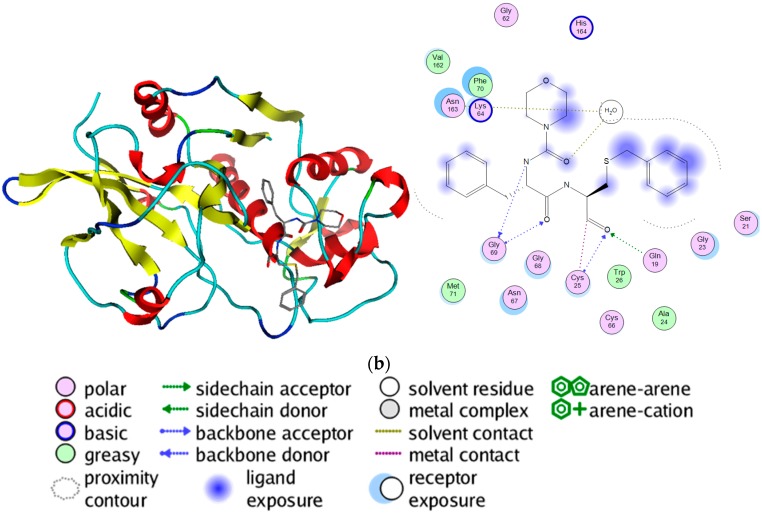
Cathepsin K and S targets. 3D structures with the corresponding 2D binding mode of bound ligands (E64 and C4P respectively) are shown for both targets (**a**) K and (**b**) S, respectively. PDB [33] codes are 1ATK [34], 1NQC [35]. The amino acid residues in 2D graph are colo-coded according to MOE defult scheme, green (hydrophobic), pink (polar, acidic, and/or basic) with arrows indicating the hydrogen bonding interactions, green arrow (sidechain donor or acceptor) and blue arrow (backbone donor or acceptor).

Cysteine cathepsins play a role in a number of diseases, including cancer, osteoarthritis, osteoporosis, autoimmune disorders and viral infection [1]. Selectivity is an important consideration in the design of inhibitors of this class of protease, especially given that many of these feature an electrophilic warhead, such as a nitrile, that interacts covalently with the active site cysteine. For instance, gene knockout studies suggest that cathepsins B (Cat B) and L2 (Cat L2) should be considered as a key anti-targets in optimization of cathepsin L (Cat L) inhibitors [2,3,4]. Cathepsin S (Cat S) is a lysosomal cysteine protease belongs to the papain superfamily, which is expressed in spleen, antigen presenting cells, such as dendritic cells, B cells, and macrophages [5]. The major role of Cat S is the processing of the major histocompatibility complex (MHC) class II associated invariant chain, which is essential for the normal functioning of the immune system. Cat S is thus an attractive therapeutic target for the treatment of autoimmune disorders. It is also reported that Cat S is implicated in various diseases such as cancer, Alzheimer’s disease, and neuropathic pain [6,7]. Other cysteine proteases, Cat K and L, play a significant role in numerous important physiological and pathological processes, such as bone resorption, cancer progression, and atherosclerosis [1,8,9,10]. Different trials were done for discovery of novel selective Cat S inhibitors, which should be safer therapeutic agents than nonselective inhibitors by avoiding off-target side effects [11,12,13,14,15,16]. Cathepsin K (Cat K) is a cysteine protease that is highly expressed by osteoclasts and has been shown to be a key enzyme involved in bone resorption [17] secreted in the extracellular acidic lacunae at the interface of the osteoclast and bone tissue, the enzyme’s primarily role consists of type I collagen degradation, one of the main constituents of bone matrix. It has been suggested that the inhibition of Cat K could slow bone resorption and it appears that Cat K represents a promising therapeutic target for the treatment of osteoporosis [18,19] (Figure 1). For a selectivity study among these targets, different methods were applied successfully to differentiate between compounds having different selectivity and were able to distinguish them from inactive database compounds [20]. Valuable tools called 2D fingerprints that can be obtained from 2D molecular graphs are extensively used for studying compound similarity and selectivity [21,22,23]. Two interesting structural fingerprints, BAPs [24] and MACCS17 [25] fingerprints, were utilized and showed good selectivity in pattern 5 analyses. The self-organizing map (SOM) principle was introduced by Kohonen in 1982 [26] which is a topographic mapping pattern recognition algorithm based on a neural network design by which objects of a multi-dimensional space are mapped into a regular predefined grid of units (neurons). This principle has been used for different tasks in chemistry and chemical biology [27,28]. Noeske *et al*., have applied a SOM algorithm for mapping known ligands according to a topological pharmacophore descriptor (CATS) and could predict potential cross-target activities [29]. Classification models using the SOM approach were designed and applied for the classification of compounds as inhibitors and non-inhibitors [30]. In addition, SOM models were used for a selectivity study of Aurora kinases [31] and HMG-Co reductase inhibitors from decoys [32]. In this work, a set of selective cathepsin K and S inhibitors of different potency was grouped and organized in a selectivity database. The goal of this study was to apply a convenient machine-learning method to study ligand-target selectivity among closely related targets through identification of potential selectivity markers in pure clusters of cathepsin inhibitors. This method utilizes SOM-based models using structural descriptors to evaluate their potential compound selectivity prediction.

## 2. Results and Discussion

### 2.1. Selectivity Database

Standard MACCS and BAPs fingerprints were used as efficient computational tools in SOM-based techniques to distinguish between compounds having different selectivity profiles. For this purpose, two previously assembled [36] data sets consisting of compounds having different selectivity profiles against two papain-like thiol proteases, including cathepsin K and S, were analyzed. The composition of these compound sets is described in Table 1. The two compound sets designated with a slash (e.g., K/S and S/K) consist exclusively of 46 and 58 compounds that are at least 50-fold more potent (*i.e*., selectivity ratio SR 50) for one target (K or S) over another (S or K), whereas the remaining compound set (e.g., KS, 49 compounds) only contains compounds with less than a 10-fold potency difference (SR < 10) for the two targets that are thus considered non-selective. These molecules cover a broad range of binding activities (PIC_50_ values between 9.7 and 3.9) and represent different chemical scaffolds. The compound data sets were collected from reported sources to evaluate the compound selectivity using a SOM classification approach.

**Table 1:** Full description of the selectivity database of cathepsin targets K and S.

Target Set	High Selectives	Low Selectives
K/S 46	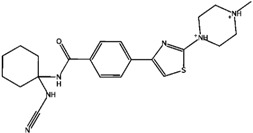 SR = 235,000	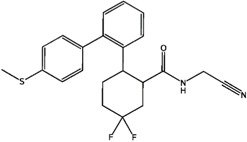 SR = 1099
KS49	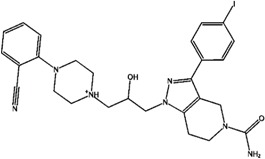 SR = 8.8	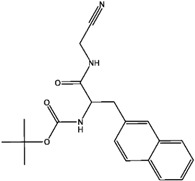 SR = 9.4
S/K 58	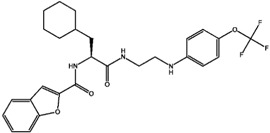 SR = 34,483	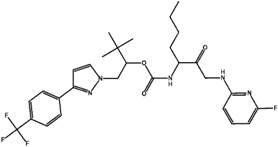 SR = 1000

The types of selectivity sets, K/S, KS, and S/K. In case of K/S set, this means we have 46 compounds which are selective for cathepsin K over S (with selectivity ratio more than 10) with corresponding two examples of high and low selective compounds. Regarding the non-selective KS group, the selectivity ratio is between 1 and not more than 10. The numbers below each molecule represent the selectivity ratio (SR), ex. 235,000 selectivity ratio for a highly selective ligand and 1099 for a low selectivity one in the case of the K/S set.

### 2.2. SOM-Based Selectivity Classification

**Figure 2:**
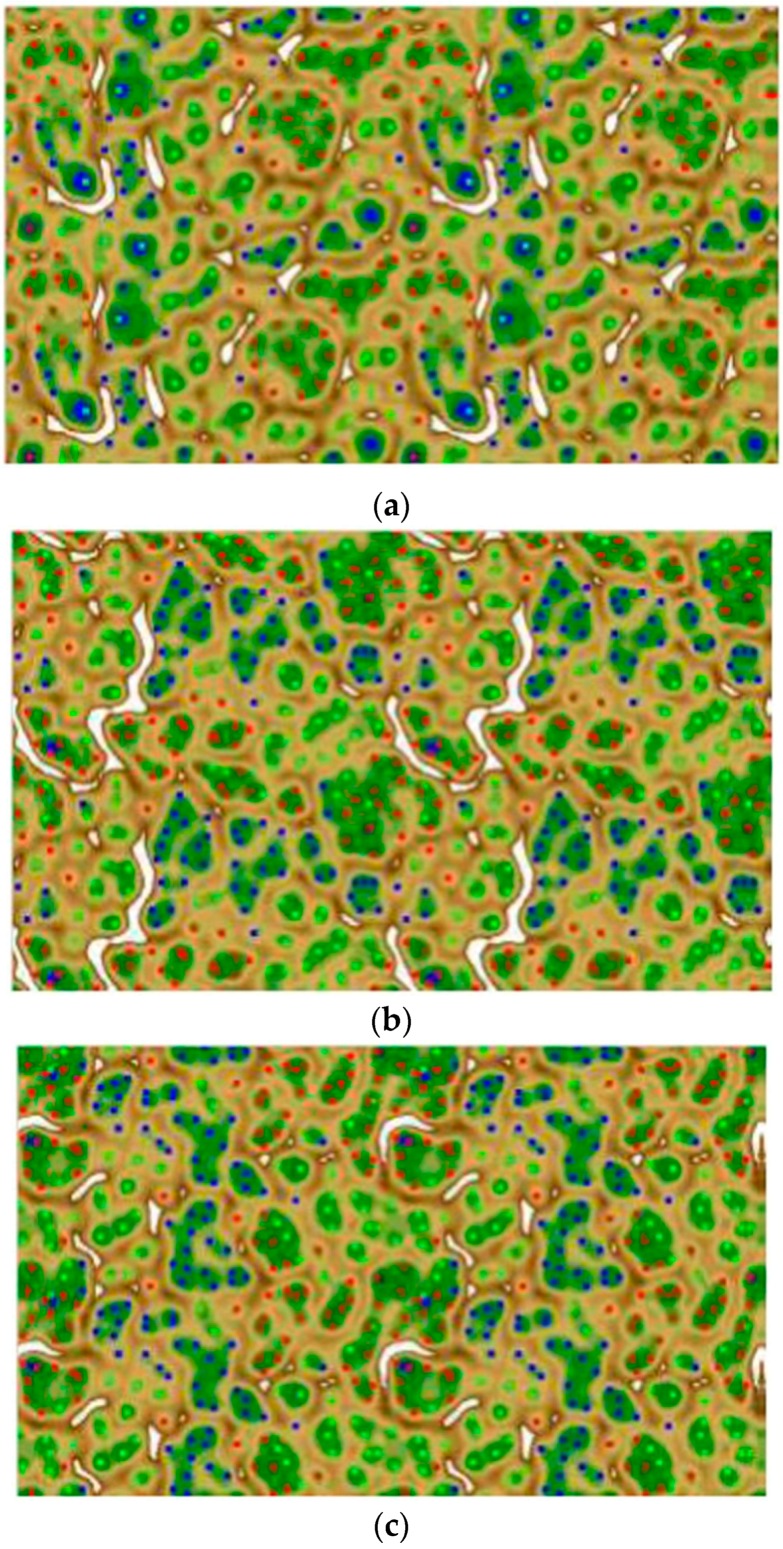
Heatmaps of the U-Matrix with the colored clusters of local regions generated with the P-Matrix. All data points with the same color belong to one cluster, red for K/S selectives, green for S/K selectives, and blue for KS non-selectives. Data points with different colors belong to different clusters. All clusters are lying in regions with small distances between the data points, and are surrounded by regions with large distances. (**a**) BAPS (**b**) MACCS and (**c**) Combined clustering.

**Figure 3:**
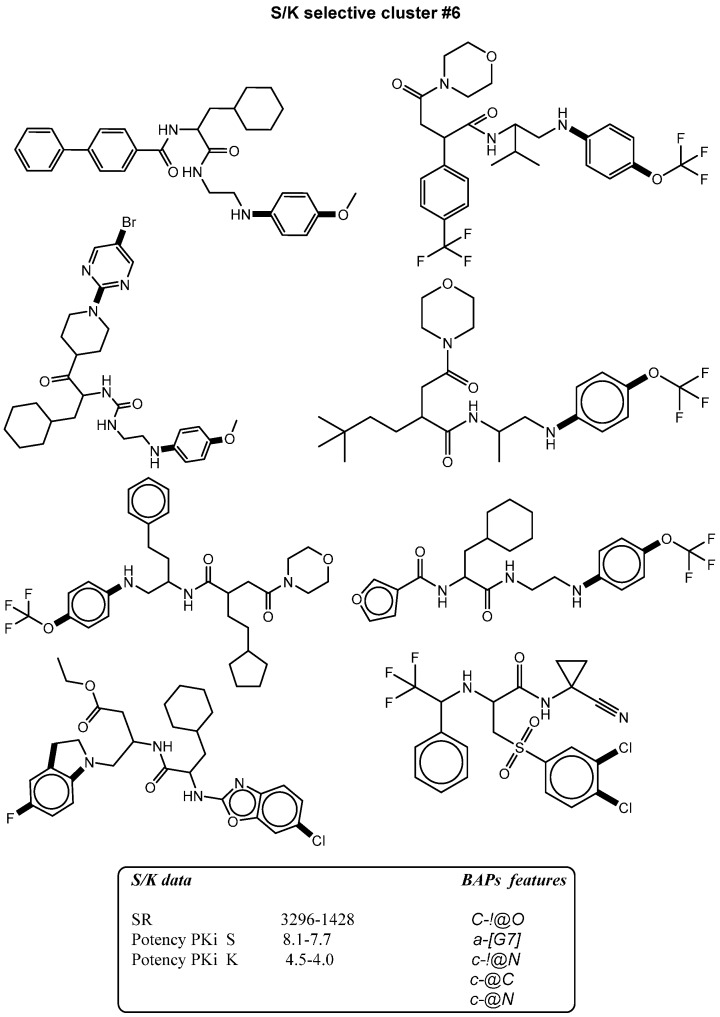
The figure shows mapping of representative BAPS selectivity signatures with high occurrence to S/K selectives presented by bold black highlighting. BAPS featuers are defined as follow, C-!@O means a aliphatic carbon atom is connected to oxygen atom in a cyclic structure by single, a-[G7] is aromatic atom attached to halogen atom, c-!@N is aromatic carbon atom connected to nitrogen atom in acyclic structure, c-@C is aromatic carbon atom connected to aliphatic carbon in cyclic structure, and c-@N is aromatic carbon attached to aliphatic nitrogen atom.

**Figure 4:**
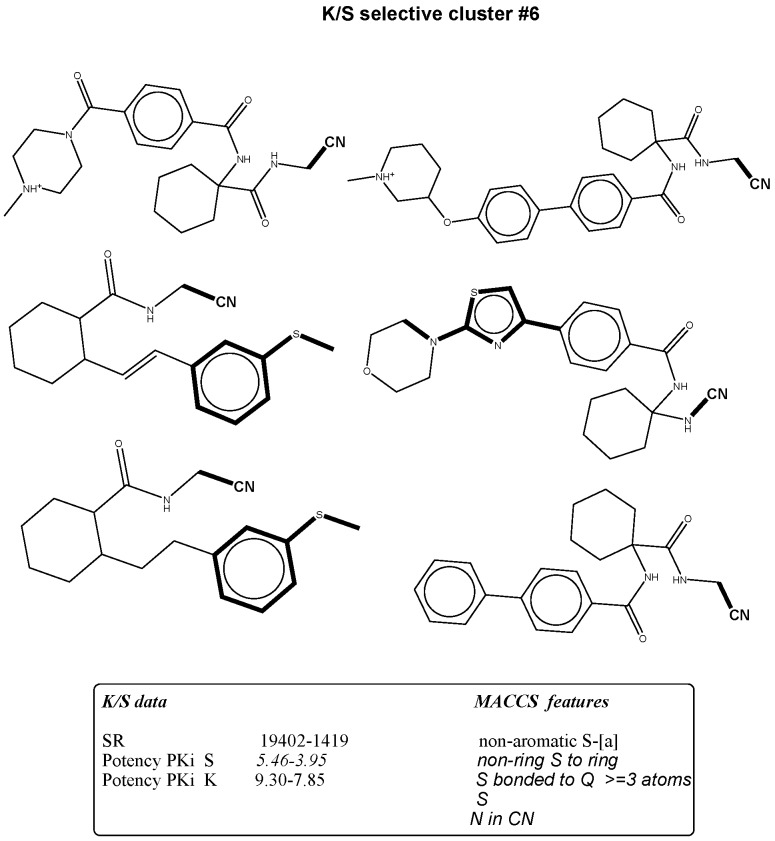
The figure shows mapping of representative MACCS selectivity signatures with high occurrence to K/S selectives presented by bold black highlighting.

**Figure 5:**
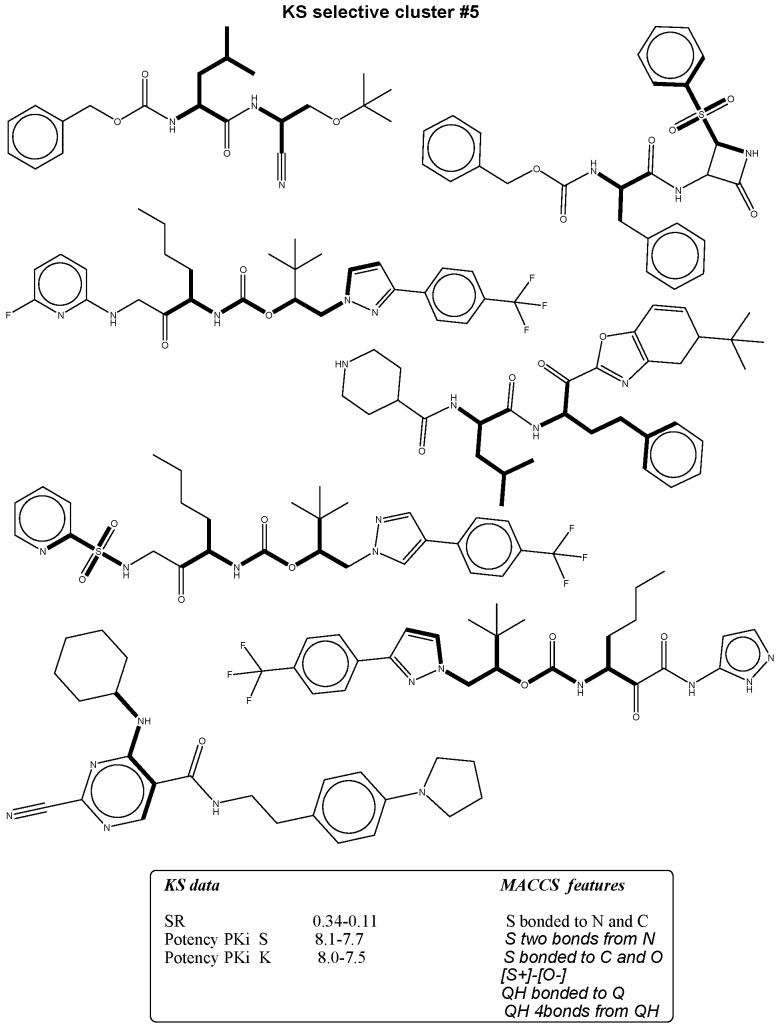
The figure shows mapping of representative MACCS selectivity signatures with high occurrence to KS non-selectives presented by bold black highlighting.

**Figure 6:**
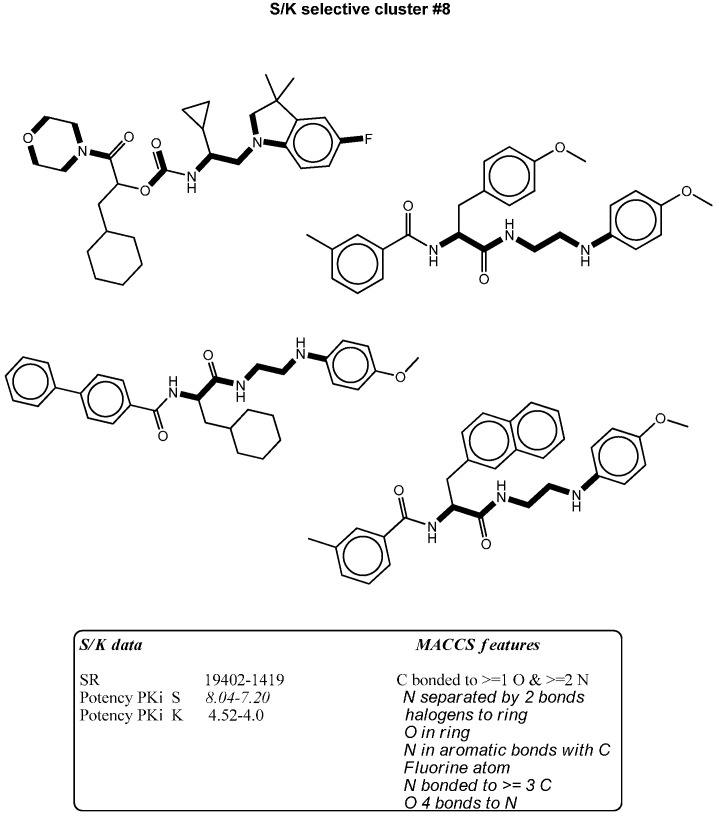
The figure shows mapping of representative MACCS selectivity signatures with high occurrence to S/K selectives presented by bold black highlighting.

The self-organizing map (SOM) principle introduced by Kohonen [26] is used widely for compound classification and clustering. It has been applied to a variety of tasks in chemistry and chemical biology ever since. In this study, the SOM algorithm was used for clustering and mapping known selective ligands according to a topological structural descriptor. The selectivity database was subjected to clustering and mapping onto a two-dimensional grid by the Emergent Self Organizing Map (SOM) approach. The ESOM method provides a nonlinear two-dimensional projection of an n-dimensional data space (chemical space), where the local neighborhood is conserved. This means, the molecules that are located close to each other on the map are also close in the original high-dimensional space. This selectivity compound library was complemented by the molecules from the ZINC database [37], a subset of 1000 molecules. This set of decoy ZINC molecules was randomly selected to challenge the classification method. For sets of selectivity compounds, each compound is converted into vectors by a fingerprint calculation. Then, the full set of data was classified using the ESOM classification. Three ESOM groups are constructed—one for classifying compounds annotated as BAPS FP, one for MACCS FP, and the third one for classifying the combination of both BAPS and MACCS. The resulting ESOM maps are further clustered to identify distinct groups of clusters with different selectivity profiles by isolation of clusters that are only composed of selective ligands (K/S, KS, and S/K) without ZINC compounds as pure selectivity clusters, Figure 2, Figure 3, Figure 4, Figure 5 and Figure 6. The Table 2, Table 3, Table 4 and Table 5 were generated for analysis of the performance of the ESOM approach. Each table shows the number of compounds, number of clusters and the purity of each cluster. In addition, the number of structural features that are highly frequent in each cluster (≥50%) are reported. The challenge in this work is clustering of compounds based on selectivity patterns with high similarity in their activity and structures. A successful model is one that could preferentially identify the target-selective compounds over the inverse selectives and non-selectives to the other targets. In general, Table 2, Table 3, Table 4 and Table 5 report the results for clusters having only compounds with selectivity obtained for MACCS, BAPS, and MACCSBAPS applications, respectively. All fingerprints successfully retrieved target-selective molecules (only compounds selective for one target) within the whole database. Depending on the selectivity set, MACCS achieved clustering of up to 25 ones with compounds ranging from 39 to singletons. BAPS achieved clustering to 26 ones and had consistently compounds ranging from greater than 27 to singletons, while the MACCSBAPS combination one does not change more than the previous types. After SOM training with 50% of selectivity database compounds, we projected the rest of compounds as test sets onto this map and analyzed the resulting distribution patterns. The two selective sets showed separate localized distributions, while the distribution of the non-selectives appears to be slightly more focused than the data (Figure 2a,b). Notably, only 6% of the two ligand classes were clustered together.

Table 1 reveals 26 clusters utilizing the BAPS descriptor with purity ranging between 100% and 50%. Twelve clusters revealed a high density of compounds only belonging to one set (2–15 compounds per cluster). Notably, seven singlets formed a seven cluster distribution, while in case of using the MACCS descriptor, a lesser number of clusters (25) with six singlets and 13 pure ones with a 7–2 compound range were revealed. More mixed clusters with purity 93%–50% appeared by using a descriptor combining both MACCS and BAPS.

In accordance, the performance of BAPS in discrimination of different selectivity sets is better than that of MACCS and the combination form (Table 5). The table indicates the number of clusters, compounds in each cluster with their types, purity, and the features that prominently appear at least with 50% more difference in frequency in compounds of the corresponding cluster to the rest of the database. The SOM was able to discriminate between the three selectivity sets. This result substantiates earlier findings that both the structural descriptors and the SOM procedure are suited for clustering compounds according to their selectivity profile. We reason that the ability of the BAPS and MACCS structural tools to recognize selective cathepsin ligands and successfully distinguish them from non-selective and inverse-selective compounds should be reflected by bit patterns that differ between these compound sets.

**Table 2:** Overall SOM performance against all cathepsins inhibitors.

FP	Number of Clusters (Purity)				Error Rate	Coverage	Correct
	Total	K/S	KS	S/K			
BAPS	26	11 (6)	8 (2)	15 (11)	18	94	76
MACCS	39	7 (4)	16 (10)	9 (5)	14	97	86
Combined	25	6 (2)	17 (10)	10 (5)	12	97	86

This shows the overall performance of SOM models. The number in brackets shows the pure cluster of corresponding data set, for example six pure clusters for K/S from a total 11 clusters using the BAPS fingerprints. Purity means the indicator of increasing pure clusters for one target set.

**Table 3:** Analysis of BAPS fingerprint performance.

Cluster #	Total	K/S	KS	S/K	Purity %	Features
1	27	2	15	10	56	4
2	16	2	11	3	69	8
3	15	15	-	-	100	8
4	12	11	-	1	92	4
5	11	-	6	5	55	7
6	8	-	-	8	100	10
7	7	6	1	-	86	6
8	6	-	4	2	67	4
9	5	-	5	-	100	8
10	4	4	-	-	100	6
11	4	-	-	4	100	6
12	4	-	-	4	100	7
13	4	-	-	4	100	5
14	3	-	-	3	100	6
15	2	1	1	-	50	6
16	2	2	-	-	100	4
17	2	-	-	2	100	6
18	2	-	-	2	100	4
19	2	-	-	2	100	5
20	1	1	-	-	100	5
21	1	-	1	-	100	4
22	1	1	-	-	100	5
23	1	-	-	1	100	6
24	1	-	-	1	100	5
25	1	1	-	-	100	3
26	1	-	-	1	100	5

The features refer to the structural bits that are frequently occurring in corresponding clusters.

**Table 4:** Analysis of MACCS fingerprint performance.

Cluster #	Total	K/S	KS	S/K	Purity %	Features
1	39	2	9	28	72	5
2	30	28	2	-	93	10
3	11	-	1	10	91	15
4	11	-	10	1	91	5
5	7	-	7	-	100	18
6	6	6	-	-	100	5
7	6	-	1	5	83	12
8	4	-	-	4	100	14
9	4	4	-	-	100	2
10	4	-	-	4	100	15
11	3	-	3	-	100	12
12	3	-	-	3	100	14
13	3	-	3	-	100	13
14	2	-	2	-	100	14
15	2	2	-	-	100	15
16	2	-	2	-	100	10
17	2	1	1	-	50	12
18	2	-	2	-	100	16
19	2	-	2	-	100	10
20	1	-	1	-	100	15
21	1	-	-	-	100	13
22	1	1	-	1	100	12
23	1	-	1	-	100	10
24	1	-	1	-	100	12
25	1	-	-	1	100	13

**Table 5:** Analysis of combined fingerprint performance.

Cluster #	Total	K/S	KS	S/K	Purity %	Features
1	30	28	2	-	93	16
2	29	-	2	20	72	7
3	12	2	7	1	92	6
4	11	-	11	10	91	17
5	8	-	1	5	62	20
6	7	-	3	-	100	7
7	7	7	-	-	100	19
8	6	-	1	5	83	15
9	6	-	-	6	100	17
10	4	-	-	4	100	14
11	4	-	4	-	100	4
12	3	4	-	-	100	15
13	3	-	3	-	100	13
14	3	-	3	-	100	18
15	2	3	-	--	50	12
16	2	1	1	-	50	14
17	2	-	2	-	100	16
18	2	-	2	-	100	12
19	1	-	-	1	100	12
20	1	-	1	-	100	20
21	1	-	1	-	100	12
22	1	-	-	1	100	15
23	1	-	1	-	100	22
24	1	-	1	-	100	15
25	1	-	-	1	100	10

### 2.3. Bit Frequency Analysis of Selectivity Clusters

The occurrence of each fingerprint bit was analyzed and computed for all clusters. A feature is defined as a selectivity marker that only occurs in at least 50% of the ligands in each cluster (Table 2, Table 3, Table 4 and Table 5). The fingerprint frequency profiles revealed that for each target pair, a varying number of MACCS bit positions were differentially set on in the selectivity sets. For example, pure cluster #3 in the BAPS results has 15 compounds of target set K/S with eight differential features, but in cluster #6, 10 features appear in eight compounds selective for S/K. Thus, bit frequency differences between selectivity sets in different pure clusters were in part substantial, which provided an explanation for the ability of BAPS and MACCS to distinguish between selective and non-selective compounds. Therefore, the structural meaning of these differential bit settings by mapping of these selectivity markers to each cluster were analyzed to identify the chemical meaning of each feature (Figure 3, Figure 4, Figure 5 and Figure 6).

### 2.4. Differential Selectivity Features Mapping

The structural features corresponding to preferentially set MACCS and BAPS keys obtained from frequency analysis of selective clusters were mapped onto the original ligands of these corresponding clusters. The results of this analysis are shown in Figure 3, Figure 4, Figure 5 and Figure 6. For example in Figure 3, characteristic bonded atom structural features including aromatic rings with different direct substituents (O, halogens, and N atoms) were found and mapped to the corresponding S/K set, while in Figure 4, different MACCS features have been identified, including aromatic atoms attached directly to a S atom and the common electrophilic CN group mapped to compounds selective for K/S. The identified features of MACCS and BAPS combinations can serve as selectivity markers and are considered characteristic of different types of cathepsin inhibitors ligands. The overall descriptions of selectivity features are characterized by the presence of aromatic atoms connected mostly to the heteroatoms O, N, and S, as well as different types of structural linkers between aromatic ring containing single or multiple amide bonds. Moreover, halogens attached to aliphatic or aromatic atoms were found in some cases. The nitrile moiety was also described as a selectivity marker in cathepsin ligands.

### 2.5. Structure-Selectivity Relationship Analysis of Cathepsin Inhibitors

**Figure 7:**
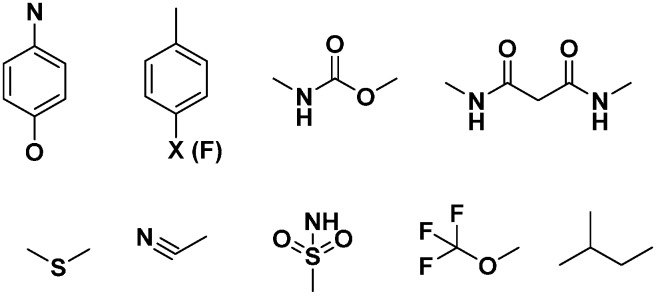
Representative selectivity features. Structural features that are commonly found in the selectivity databases based on SOM and fingerprint analysis.

Based on the SOM results, the structure-selectivity relationships (SSRs) of the selectivity sets of cathepsin inhibitors, are summarized in Figure 7. Common structural features are presented according to the structures and fingerprint analyses of cathepsin inhibitors, including substituted heterocyclic rings with amino, halide, and carbon functionalities. In addition, linkers occur among the aromatic or hetreroaromatic nuclei have one or multiple amide or ester bonds or both (carbamate). Moreover, different substituents are formed of alkyl or haloalkyl chains (mainly flouro). One structural fragment commonly present is the electrophile nitrile moiety that aids in covalent binding of ligands. In addition, Sulphur-containing fragments like thioether and sulphonamide were found. These structural features may be helpful in interpretation of ligand selectivity and useful for medicinal chemists to design new selective inhibitors of cathepsins.

### 2.6. Experimental Validation of Clustering

**Figure 8:**
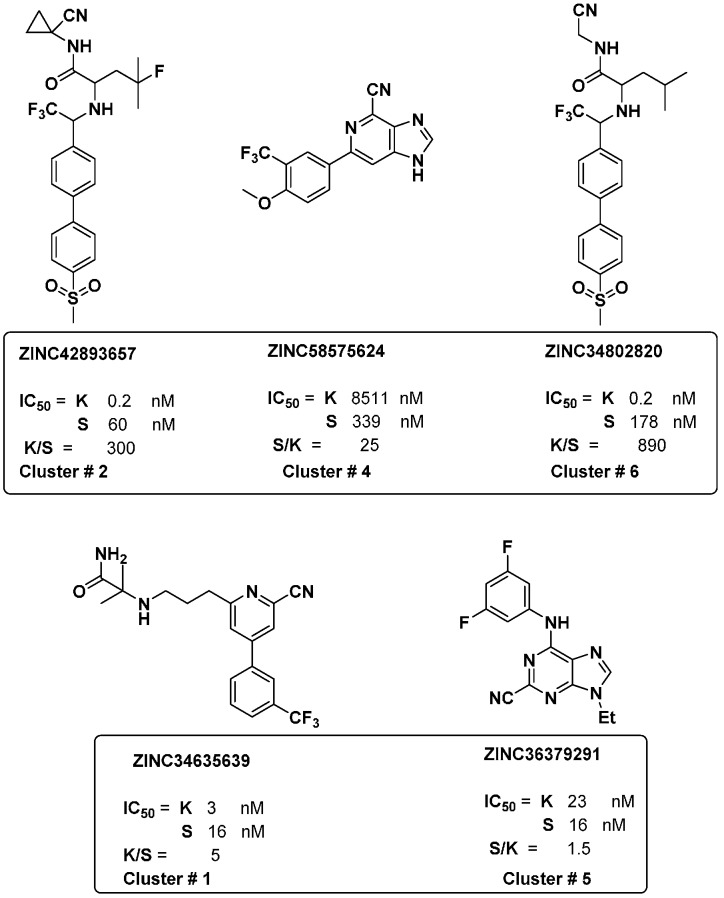
New cathepsin set. The figure shows retrieved ZINC compounds predicted and taken from selective clusters. The IC_50_ of these compounds and clusters are reported with corresponding calculated selectivity.

To validate the accuracy and robustness of the stability prediction of the SOM model, a different external subset of the ZINC database (1000 compound) [37] was used and merged with the original selectivity database for searching for selective cathepsin inhibitors. These compounds were selected based upon their high degree of similarity to selective ligands for searching for novel selective cathepsin inhibitors. Then, the same protocol was applied by calculation of fingerprint descriptors and utilizing all data in the SOM approach. The clusters were identified and ZINC molecules were selected from each selective pure cluster that was close to the reference selectivity ligands. Among these ZINC structures, five compounds were analyzed based on their chemical structure and mapped with selectivity features. In accordance, the activity profile of such new found ZINC hits was reported and the corresponding selectivity ratios were calculated. It was seen that the activity against cathepsin K and S ranges between 0.2 nM to 8511 nM. In addition, the compounds revealed different degrees of selectivity, as two compounds are selective for K over S (300, 890), one compound is selective for S over K (25), and two non-selective compounds (5, 1.5) were found (Figure 8) [37,38,39,40,41,42,43].

Interestingly, the reported activity/selectivity profiles of these novel hits confirmed our method for addressing ligand selectivity and the success of using two structural fingerprints in defining and distinguishing cathepsin inhibitors of different selectivity profiles.

## 3. Materials and Methods

### 3.1. Selectivity Database

A dataset of 153 selective cathepsin inhibitors exhibiting different selectivity profiles was collected from the literature and databases [36]. On the basis of systematic compound evaluation, a total of 153 different molecules were organized into three selectivity sets, as described in Table 1. Each set of selective (K/S & S/K) compounds consists of compounds that were selective for one target over a closely related one (with at least 50-fold difference in potency), whereas compounds in the non-selective subset (KS) showed comparable potency against both targets. The number of compounds per set ranged from 46 to 59 compounds between selective and non-selective compounds (Table 1).

### 3.2. Compound Structures and Fingerprint Representation

The molecular structures of the current database were built and cross-checked using the builder in the MOE software [44]. Each molecule in the database was optimized using the molecular mechanics force field which implemented in MOE. 2D fingerprint calculations of different structural designs was performed for all the compounds. These fingerprints are Molecular ACCess System (MACCS), consisting of 166 bits [25] and Bonded Atom Pairs (BAPS), consisting of 117 bits [24]. The merged fingerprint (MACCSBAPS) was built by combination of both typical MACCS and BAPS. The output files were saved and further used as inputs for the SOM application step.

### 3.3. SOM and ESOM Neural Networks

**Figure 9:**
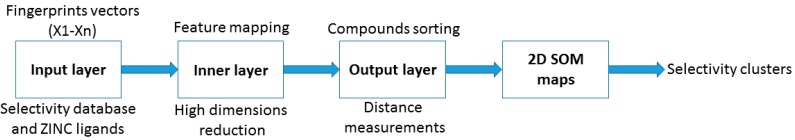
Chart for the generation of molecular selectivity SOM maps.

SOM has attracted the attention of researchers because of its ability to analyze complex multidimensional data in an intuitively comprehensible visual manner. The SOM technique can be used well in compound pattern recognition, combinatorial library comparison, and combinatorial library design, splitting a dataset into the proper training and test sets before constructing a (Quantitative Structural-Activity Relationship (QSAR) model and other studies which require the analysis of distributions of compounds in some chemical space [31]. The ESOM software [26] was used for performing Kohonen’s SOM. Kohonen’s SOM has the special property of effectively creating a spatially organized internal representation of various features of input signals and their abstractions. In a SOM, the neurons are arranged in a two dimensional array to generate a two-dimensional feature map such that similarity in the data is preserved. In other words, if two input data vectors are similar, they will be mapped into the same neuron or closely together in the two-dimensional map. Data with similar input were mapped into the same neuron or neighbor neurons in the two-dimensional map, Figure 9. Herein, SOM was applied to split the data set into a training set and a test set, and also used as one method to develop classification models to classify the selectivity of cathepsin inhibitors. To visualize data of multiple dimensions, a projection from the high-dimensional space onto two dimensions is needed. There are many algorithms which project a high-dimensional data space into two or three dimensions like PCA and ICA for linear projections and MDS and Sammon’s mapping for nonlinear projections. The emergent self-organizing map (ESOM) is a projection onto a grid of neurons, called map. Emergent SOM (ESOM) is a variation of SOM, which handles a larger number of neurons (at least 4000) and uses boundless maps [26,45]. It embeds the maps to a finite boundless space such as sphere or toroid. In the ESOM approach, two visualization methods of the ESOM maps are used, namely, the P-matrix and the U-matrix and a topological correct ESOM projects a cluster onto a coherent area on the map (cluster area). Points within the cluster are mapped to the inside of the cluster area. Data points at the border (Surface) of the cluster are projected to the border of the cluster area. The P-matrix visualizes the density in the input data space using the Pareto density estimation. In general, it is suitable for dealing with slowly changing densities and overlapping clusters. The U-matrix visualizes neurons on an ESOM map by a color coding that represents the sum of distances to all immediate neighbors normalized by the largest value in the neighboring neurons. Generally, the U-matrix is appropriate for handling data points which are clearly separated from each other. The ESOM program is available at http://databionic esom.sourceforge.net/. The original paper by Ultsch describes the general ESOM training procedure in details. The advantage of SOM/ESOM is that it is able to provide an intuitive visualization of the similarity of input data [46,47].

### 3.4. Bit Frequency Analysis and Feature Mapping

Fingerprint bits of MACCS and BAPS types were analyzed by calculation of the frequency of occurrence in each cluster. The feature of at least 50% occurrence was selected and identified as selectivity markers. This analysis was carried out with an in-house generated perl script.

### 3.5. Experimental Validation of Clustering

The SOM models was experimentally validated using a 1000 compound subset of the ZINC database randomly selected, but having more structural similarity to the selectivity database. The compounds in ZINC was extracted and merged with the selective compounds and utilized for SOM clustering. The given clusters were analyzed and only mixed clusters formed of ligands of specific annotation (K/S, KS, or S/K) with ZINC compounds were taken. Five compounds were selected as nearest neighbors for selectivity database compounds from different clusters and were searched for their activity profile in web databases supporting biological activity.

## 4. Conclusions

Cathepsin enzyme targets are of increasing interest due to their involvement in extracellular proteolytic activities and regulation of intermediates in certain diseases. A special selectivity database for two cathepsins was built and fully characterized. In this work, emergent SOMs were calculated using an ESOM algorithm with multiple neurons for selectivity set clustering. Two structural molecular descriptors, BAPs and MACCS fingerprints, were selected to be utilized in selectivity prediction of cathepsin K and S inhibitors by the self-organizing map technique. The resulting maps and clusters have extensively been analyzed. Investigation of the performances of fingerprints and extensive structure-selectivity relationship analysis of compound clusters led to the extraction of several selective substructures that are important in interpretation of inhibitor selectivity. These selectivity markers provided by fingerprint analysis could be very helpful in the lead optimization or the design of new hits with better activity and selectivity towards cathepsin targets. Finally, successful SOM-based selectivity clustering was applied and potential cathepsin K and S inhibitors were predicted.

## Abbreviations

The following abbreviations are used in this manuscript:

### Abbreviations

ESOM: Emergent Self Organizing Map
MACCS: Molecular ACCess System
BAPs: Bonded Atom Pairs
PCA: Principal Component Analysis
